# Association between Diabetes Complications and the Triglyceride-Glucose Index in Hospitalized Patients with Type 2 Diabetes

**DOI:** 10.1155/2021/8757996

**Published:** 2021-10-11

**Authors:** Ying Pan, Shao Zhong, Kaixin Zhou, Zijian Tian, Fei Chen, Ziqing Liu, Zhaoxu Geng, Shaoyun Li, Rong Huang, Heyou Wang, Weiqing Zou, Ji Hu

**Affiliations:** ^1^Department of Endocrinology, The Second Affiliated Hospital of Soochow University, Suzhou, Jiangsu, China; ^2^Department of Endocrinology, Kunshan Hospital Affiliated to Jiangsu University, Kunshan, Jiangsu, China; ^3^College of Life Sciences, University of Chinese Academy of Sciences, Beijing, China; ^4^Institute of Biophysics, Chinese Academy of Sciences, Beijing, China; ^5^Big Data Laboratory, Renji Hospital, University of Chinese Academy of Sciences, Chongqing, China; ^6^Bioland Laboratory (Guangzhou Regenerative Medicine and Health Guangdong Laboratory), Guangzhou, China; ^7^Penglang Community Health Service Center, Kunshan, Jiangsu, China

## Abstract

**Background:**

Triglyceride-glucose (TyG) index is a convenient indicator of insulin resistance. It has been shown to be associated with macrovascular and microvascular complications in nonhospitalized diabetic patients. However, whether TyG index is a risk factor of diabetes vascular complications in hospitalized type 2 diabetic patients is unclear. We sought to explore the association between TyG index and the risk of macrovascular and microvascular complications in a large Chinese cohort of hospitalized patients.

**Method:**

A total of 4,721 patients with type 2 diabetes (T2D) who were hospitalized in the Department of Endocrinology, Kunshan Hospital Affiliated to Jiangsu University were enrolled between January 2015 and November 2020. TyG index was calculated as ln[fasting triglycerides (mg/dL) × fasting glucose (mg/dL)/2]. Measures of macrovascular complications included brachial-ankle pulse wave velocity (ba-PWV) and ankle-brachial index (ABI), whilst urine microalbumin (MAU), chronic kidney disease (CKD), and diabetic retinopathy (DR) were evaluated for microvascular complications. Logistic regressions were used to examine the association between TyG index and diabetes complications.

**Results:**

In univariate logistic regressions, higher TyG index was significantly (*p* < 0.002) associated with increased odds of MAU (OR = 1.39, 95% CI: [1.22~1.59]) and ABI (OR = 1.31, 95% CI: [1.10-1.57]) but not CKD, DR, or ba-PWV. After controlling for confounders such as age, sex, and body mass index (BMI), TyG index remained strongly (*p* < 0.002) associated with MAU and ABI. These associations were more pronounced (*p* < 0.001) in patients with poor glycemic control or in the elderly.

**Conclusion:**

Hospitalized patients with an elevated TyG index were at a higher risk of lower limb vascular stenosis and nephric microvascular damage. Close monitoring of TyG index in patients with younger age or poor glycemic control could potentially reduce the burden of diabetes complications and prevent readmission.

## 1. Introduction

Insulin resistance is a manifestation of metabolic abnormalities, also known to be one of the main pathogenic drivers of type 2 diabetes (T2D) [1]. Chronic hyperglycemia and insulin resistance could lead to vascular damage and are regulated by multiple pathophysiological processes [2]. Therefore, monitoring insulin resistance is of great significance for the prevention and treatment of T2D and its complications.

The gold standard method to measure insulin resistance is through euglycemic hyperinsulinemic clamp. However, it was rarely conducted in clinical settings due to its complex process and high costs [3]. Triglyceride-glucose (TyG) index had been shown to be significantly associated with insulin resistance in previous studies and proposed as a proxy for insulin resistance [4]. TyG index could be calculated from routinely collected serum biochemical parameters and readily available for clinical application widely. It has been shown that TyG index was significantly elevated in patients with T2D and could be used to identify individuals at high risk for early prevention [5]. Another investigation reported that increased level of TyG index was significantly associated with higher risk of arterial stiffness and nephric microvascular damage in a Chinese elderly community population, raising the potential to adopt TyG index for evaluating vascular risk of diabetic complication in outpatient settings [6].

However, the serum glucose and lipid levels of hospitalized patients with T2D were often relatively high. They were metabolically unstable and often coupled with other acute illness. It is unknown whether TyG index remains a strong risk predictor of diabetes complications in this group of patients with high incidence rate of chronic complications. In this study, we assembled a large group of hospitalized patients with T2D and investigated whether TyG index was associated with a wide range of macrovascular and microvascular complications as recorded in the electronic medical record system.

## 2. Method

### 2.1. Participants

A total of 4,721 type 2 diabetic patients who were hospitalized in the Department of Endocrinology, Kunshan Hospital Affiliated to Jiangsu University between January 2015 and November 2020 were enrolled. Participants were excluded if they (1) were 18 years or younger; (2) were diagnosed type 1 diabetes or other special types of diabetes; (3) were suffering from acute complications of diabetes, severe chronic complications, or malignant tumors; and (4) had a diagnosis of urinary tract infection, renal calculi, or primary renal disease. The protocol was approved by the institutional review board of the hospital, and all enrolled patients gave written informed consent.

### 2.2. Clinical Information Collection

Data for all the patients were retrieved from the inpatient medical record system of the Kunshan Hospital Affiliated to Jiangsu University. Essential information such as sex, age, medical history, and smoking status were recorded at admission. Demographic characteristics such as height, weight, and waist circumference of the patients were taken by the nurses. Venous blood samples were collected at the second morning of admission after fasting for 8-10 hours. Biochemical parameters such as fasting blood glucose (FBG), glycated hemoglobin (HbA1c), uric acid (UA), creatinine (Cr), total cholesterol (TC), triglyceride (TG), high-density lipoprotein cholesterol (HDL-C), and low-density lipoprotein cholesterol (LDL-C) were measured with the venous blood sample. The estimated glomerular filtration rate (eGFR) was calculated from the Epidemiology Collaboration (CKD-EPI) equation [7]. The TyG index was calculated as ln[fasting triglycerides (mg/dL) × fasting glucose (mg/dL)/2].

### 2.3. Assessment of Macrovascular and Microvascular Complications

For macrovascular complications, the brachial to ankle pulse wave velocity (ba-PWV > 1800 cm/s) was used to assess atherosclerosis, and the ankle-brachial index (ABI<0.9) was used to assess lower limb vascular stenosis. For microvascular complications, chronic kidney disease (CKD) was defined as eGFR ≤ 60 mL/min per 1.73 m^2^, microalbuminuria (MAU) was defined as urinary albumin to creatinine ratio (UACR ≥ 30 mg/g), and diabetic retinopathy (DR) was defined as any of the following diagnosis: microaneurysm formation, retinal hemorrhage, hard exudation, cotton flocculus, retinal microvascular abnormalities, venous beading, retinal neovascularization, vitreous hemorrhage, and fibrous hyperplasia. The Omron Non-Invasive Vascular Screening Device (BP-203RPEIII) was used to measure blood pressure, ba-PWV, and ABI. DR was assessed by retinal images captured by TOPCON TRC.NW400 nonmydriatic retinal camera, which were then analyzed and diagnosed by experienced clinicians.

### 2.4. Statistical Analysis

Quantitative parameters were shown as the mean ± standard deviation, and qualitative parameters were presented as numbers with the percentages in parentheses. The quantitative parameters of males and females were compared by Student's *t*-test, and qualitative parameters were compared by the chi squared test. We used Pearson's correlation to assess the association between the TyG index and cardiovascular risk factors. We used logistic regression to assess the relationship between the TyG index and indicators of macrovascular and microvascular complications. We further conducted subgroup analyses according to gender, age, and HbA1c levels and stratified the patients by quartiles of TyG index. Statistical analyses were performed using *R* studio software version 1.2.1335. The statistical significance level was set at *p* < 0.05.

## 3. Result

### 3.1. Basic Characteristics of the Participants

As shown in Table 1, a total of 4,721 hospitalized patients with T2D were enrolled in this study, including 2,192 women and 2,529 men. Compared with women, men had higher levels of HbA1c, diastolic blood pressure (DBP) and eGFR, but lower levels of systolic blood pressure (SBP), HDL-c, LDL-c, TC, ba-PWV, and were younger at the time of admission (all *p* < 0.001). A higher incidence rate of lower limb vascular stenosis and a lower incidence rate of microalbuminuria (both *p* < 0.05) were observed in men as compared to those in women.

### 3.2. TyG Index Correlation with Cardiovascular Risk Factors

We first examined the association between TyG index and established cardiovascular risk factors. As shown in Table 2, TyG index was positively correlated with SBP (*r* = 0.041), DBP (*r* = 0.148), BMI (*r* = 0.246), LDL-c (*r* = 0.46), and TC (*r* = 0.362) and negatively correlated with age (*r* = −0.181) and HDL-c (*r* = −0.315) (*p* < 0.05 for all). In keeping with previous studies, higher TyG index was associated with increased cardiovascular risk, indicating that TyG index remained a valid metabolic index among those hospitalized patients with T2D.

### 3.3. TyG Index Association with Macrovascular and Microvascular Complications

We then used logistic regression to examine the association between TyG index and common complications in the whole sample. As shown in Table 3, the univariate logistic regressions showed that higher TyG index was significantly associated with increased risk of MAU (OR = 1.39, 95% CI: 1.22-1.59) and ABI (OR = 1.31, 95% CI: 1.10-1.57), but not CKD, DR, or ba-PWV. After controlling for confounders such as age, sex, BMI, and smoking status, the multivariate logistic regressions showed that MAU (OR = 1.48, 95% CI: 1.26-1.73) and ABI (OR = 1.42, 95% CI: 1.14-1.76) remained strongly associated with increased TyG index, indicating that TyG index was significantly associated with MAU and ABI in hospitalized T2D patients.

### 3.4. Sensitivity Analysis

We next performed sensitivity analyses to further explore the context in which the TyG index was associated with MAU and ABI. As shown in Table 4, both MAU and ABI remained strongly associated with TyG index when patients were stratified by gender. The associations between TyG index and MAU and ABI were stronger in the younger patients as compared to those aged over 60, suggesting in these physically fragile patients TyG index was less correlated with diabetes complications. When patients were stratified by the median HbA1c of 8.6%, weaker associations were observed in patients with better glycemic control compared to those with poor glycemic control, indicating insulin resistance was less correlated with diabetes complications among metabolically unstable patients.

### 3.5. Logistic Regression according to the Quartiles of TyG Index

As shown in Table 5, the patients were then divided into four groups Q1, Q2, Q3, and Q4 according to the quartiles of their TyG index levels. With Q1 serving as the reference, multivariate logistic regressions adjusting for age, gender, HbA1c, smoking habit, and BMI were conducted. The results showed that the risks in the Q4 group of MAU (OR = 1.73, 95%CI = 1.28 − 2.35) and ABI (OR = 1.63, 95%CI = 1.10 − 2.43) were significantly higher than they were in the Q1 group (*p* < 0.01) in univariate analyses. After adjusting for confounders, risks in the Q4 group of MAU (OR = 1.80, 95%CI = 1.28 − 2.56) and ABI (OR = 1.66, 95% CI = 1.03 − 2.71) were still significantly higher than they were in the Q1 group (*p* < 0.05). That further proved that an increased TyG index would lead to macrovascular and microvascular complications.

## 4. Discussion

In this study, the primary finding was that TyG index was highly correlated with UACR (>30 mg/g) and ABI (<0.9) in hospitalized patients with T2D. These associations with ABI and MAU were more pronounced in patients with poorly controlled glucose and in the elderly. We did not observe significant associations between TyG index and other microvascular or macrovascular complications such as DR, CKD and ba-PWV.

Patients with T2D could be hospitalized for many reasons other than metabolic illness. The participants in our study were admitted to this community-based teaching hospital primarily due to metabolic reasons such as poor glycemic control, the onset, or fast deterioration of diabetic complications. Therefore, the association analyses performed in this cohort would be less confounded by other acute illness. Compared to the general population with T2D, these hospitalized patients with T2D had higher levels of TyG index and vascular complications and were later in the disease course. The large number of participants recruited over 5 years made the sample representative of various metabolic states and enabled the exploration of the correlations between TyG index and multiple vascular outcomes.

Diabetic nephropathy is a multifactorial disease and represents a major microvascular complication for individuals with diabetes. Our results showed that TyG index was significantly correlated with MAU but had no significant correlation with CKD. This is in line with a previous study showing that TyG index, as compared with HOMA2-IR, had a stronger correlation with urinary MAU but had no obvious correlation with eGFR [8]. These results suggested that the effect of insulin resistance on diabetic nephropathy was stronger in the early stage than in the late stage when renal function insufficiency occurs. The possible explanation underlying this phenomenon might be that the early initiation of the renal function changes in diabetic patients was evoked by the manifestations of insulin resistance, including hyperglycemia and dysfunction of lipid oxidation and utilization. With the progress of dysglycemia, other complex metabolic abnormalities such as hyperglycemia triggered metabolic acidosis and persistence inflammatory reaction might occur on top of insulin resistance and post huge burden to the vulnerable kidney resulting in the exacerbation of renal dysfunction [9, 10]. More studies should be exclusively carried out to clarify the mechanisms contributing to the progress of renal insufficiency in this later stage.

Macrovascular complications are the main cause of death in T2D patients. It has also been reported that peripheral arterial disease was associated with the risk of amputation in T2D patients [11]. Cumulative evidence suggest that insulin resistance causes overproduction of reactive oxygen species, which could in turn lead to endothelial dysfunction and inflammation, and play a major role in precipitating diabetic vascular disease [12]. One previous study showed that TyG index was significantly correlated with PWV and macrovascular damage in a community-based cohort [13]. Here, we found significant association between TyG index and ABI, but not with ba-PWV. The association with ABI was stronger in the HbA1c > 8.6%group who were more likely to be in an advanced stage of diabetes course. This is in line with the observation that ABI was a better marker of peripheral vascular impairment in more advanced atherosclerotic stage whilst ba-PWV characterizes coronary atherosclerosis more closely. However, our multivariate analysis did find a significant (*p* < 0.001) association between ba-PWV and TyG index, suggesting that risk of TyG index on macrovascular complications could be confounded by other clinical factors such as age and BMI.

It is interesting our sensitivity analysis found that among younger patients (age < 60), TyG index was more associated with MAU and ABI. This probably reflects the fact that the early onset of T2D tends to be more driven through beta cell functional impairment and insulin resistance, whilst diabetes in the elderly are more related to the aging process. Similarly, TyG index was more associated with ABI and MAU in the group with poor glycemic control. Our grouped TyG index association analyses demonstrated TyG index level had a dosing impact on the increased risk of ABI and MAU. Therefore, patients with younger age, high HbA1c, and high TyG index at admission should be monitored more closely on their microvascular and macrovascular complications. Given that preventing readmission is a key component of diabetes care for inpatients, TyG index could also serve as the convenient surrogate of insulin resistance to form a structured discharge plan for those patients with high risk of complications.

This study recruited patients from a single hospital. Although the suburban population it serves largely represents the general Chinese population, caution should still be taken while interpreting the results. There were sparse measures of HOMA-IR (Homeostasis Model Assessment for Insulin Resistance) available in our observational inpatient data, and we were only able to show that TyG index was a valid proxy of insulin resistance by demonstrating its associations with established cardiovascular risk factors. Moreover, compared to the outpatient setting in which TyG index could be affected by diet and exercises, TyG index measurements in the hospital setting could also be confounded by the treatments with hypoglycemic agents and lipid lowering drugs. Therefore, it would require carefully designed clinical trial to evaluate the correlation between TyG index and insulin resistance in an extended period of time. Due to the nature of our cross-sectional data, this study is limited in what we can infer about the causality of the results. A prospective cohort study would be required to evaluate TyG index's predicting ability regarding macrovascular and microvascular complications.

Although limited data on HOMA-IR were available in this observational cohort, the strong correlations between TyG index and cardiovascular risk factors demonstrated that it remains a valid proxy of insulin resistance.

## 5. Conclusions

An elevated TyG index was associated with higher risk of lower limb vascular stenosis and nephric microvascular damage in hospitalized T2D patients. As a low-cost, routinely available proxy for insulin resistance, TyG index would be useful to identify younger hospitalized patients with high HbA1c for more complication screening and inform structured discharge plan to prevent readmission.

## Figures and Tables

**Table 1:** Basic characteristics of the T2D patients.

Variables	Total (*n* = 4721)	Female (*n* = 2192)	Male (*n* = 2529)	*p* value
Age	59.56 ± 13.02	62.45 ± 11.74	57.06 ± 13.54	<0.001
FBG (mmol/L)	9.05 ± 3.35	8.92 ± 3.33	9.16 ± 3.37	0.022
HbA1c (%)	9.00 ± 2.22	8.81 ± 2.08	9.17 ± 2.32	<0.001
BMI (kg/m^2^)	25.30 ± 3.64	25.32 ± 3.80	25.28 ± 3.50	0.370
SBP (mmHg)	136.83 ± 19.38	138.08 ± 19.56	135.74 ± 19.16	<0.001
DBP (mmHg)	77.07 ± 10.97	75.18 ± 10.01	78.70 ± 11.50	<0.001
HDL-c(mmol/L)	1.24 ± 0.29	1.32 ± 0.30	1.17 ± 0.27	<0.001
LDL-c (mmol/L)	2.69 ± 0.91	2.72 ± 0.91	2.66 ± 0.91	0.139
TC (mmol/L)	4.30 ± 1.09	4.42 ± 1.08	4.19 ± 1.09	<0.001
TG (mmol/L)	1.83 ± 1.69	1.79 ± 1.48	1.86 ± 1.86	0.356
eGFR (mL/min/1.73m^2^)	96.74 ± 22.96	94.05 ± 22.93	99.10 ± 22.73	<0.001
DR, *n* (%)	1095 (37.29%, *n* = 2936)	505 (38.87%, *n* = 1299)	590 (36.04%, *n* = 1637)	0.124
ABI < 0.9, *n* (%)	377 (8.16%, *n* = 4622)	154 (7.20%, *n* = 2140)	223 (8.98%, *n* = 2482)	0.031
UACR ≥ 30 mg/g (%)	445 (14.99%, *n* = 2968)	177 (13.06%, *n* = 1355)	268 (10.60%, *n* = 1613)	<0.001
ba-PWV (m/s)	1600 ± 437	1658 ± 467	1550 ± 403	<0.001
TyG	9.20 ± 0.78	9.19 ± 0.76	9.21 ± 0.80	0.853

FBG: fasting blood glucose; HbA1c: glycosylated hemoglobin; BMI: body mass index; SBP: systolic blood pressure; DBP: diastolic blood pressure; HDL-C: high-density lipoprotein cholesterol; LDL-C: low-density lipoprotein cholesterol,; TC: total cholesterol; TG: triglyceride; eGFR: estimated glomerular filtration rate; DR: diabetic retinopathy; ABI: ankle brachial index; UACR: urine albumin to creatinine ratio; ba-PWV: brachial to ankle pulse wave velocity; TyG: triglyceride glucose index.

**Table 2:** The correlation between TyG index and cardiometabolic risk factors.

	*r*	*p* value
Age	-0.181	<0.001
Sex	0.003	0.853
SBP	0.041	0.018
DBP	0.148	<0.001
BMI	0.246	<0.001
LDL-c	0.460	<0.001
HDL-c	-0.315	<0.001
TC	0.362	<0.001

TyG: triglyceride glucose; SBP: systolic blood pressure; DBP: diastolic blood pressure; BMI: body mass index; HDL-C: high-density lipoprotein cholesterol; LDL-C: low-density lipoprotein cholesterol; TC: total cholesterol.

**Table 3:** Logistic regression analyses of TyG index on diabetic complications.

	Univariate		Multivariate	
	OR	*p* value	OR	*p* value
CKD (eGFR<60 mL/min per 1.73 m^2^)	1.06 (0.92-1.21)	0.430	1.02 (0.87-1.21)	0.783
MAU(UACR≥30 mg/g)	1.39 (1.22-1.59)	<0.001	1.48 (1.26-1.73)	<0.001
DR	1.03 (0.93-1.14)	0.531	0.96 (0.85-1.08)	0.490
ABI < 0.9	1.32 (1.10-1.57)	0.002	1.42 (1.14-1.76)	0.002
ba − PWV > 1800 cm/s	0.99 (0.90-1.08)	0.776	1.38 (1.21-1.57)	<0.001

CKD: chronic kidney disease; MAU: microalbuminuria,; ABI: ankle–brachial index; ba-PWV: brachial-ankle pulse wave velocity; DR: diabetic retinopathy.

**Table 4:** Sensitivity analyses of the association between TyG and MAU and ABI when patients were stratified by sex, age, and HbA1c.

	MAU		ABI	
	OR	*p* value	OR	*p* value
Male	1.39 (1.17-1.65)	<0.001	1.3 (1.04-1.62)	0.02
Female	1.38 (1.11-1.70)	0.003	1.34 (1-1.78)	0.04
Age < 60	1.6 (1.31-1.96)	<0.001	1.94 (1.43-2.61)	<0.001
Age ≥ 60	1.29 (1.07-1.56)	0.007	1.23 (0.98-1.55)	0.07
HbA1c < 8.6	1.26 (1.04-1.53)	0.010	1.04 (0.76-1.40)	0.82
HbA1c ≥ 8.6	1.49 (1.20-1.85)	<0.001	1.62 (1.26-2.08)	<0.001

**Table 5:** Univariate and multivariate logistic regressions of MAU and ABI by TyG quartiles.

	Univariate		Multivariate	
	MAU	ABI	MAU	ABI
Q1 (6.21 ≤ TyG < 8.67)	1	1	1	1
Q2 (8.67 ≤ TyG < 9.17)	1.12 (0.81-1.55)	1.00 (0.65-1.55)	1.05 (0.74-1.50)	1.04 (0.64-1.71)
Q3 (9.17 ≤ TyG < 9.67)	1.74 (1.29-2.37)	1.35 (0.90-2.05)	1.75 (1.25-2.45)	1.24 (0.77-2.03)
Q4 (9.67 ≤ TyG ≤ 12.87)	1.73 (1.28-2.35)	1.63 (1.10-2.43)	1.80 (1.28-2.56)	1.66 (1.03-2.71)
*p* for trend	<0.001	0.005	<0.001	0.026

## Data Availability

The electronic medical record data retrieved from the Kunshan hospital was anonymized for this study. Summary data that were used to support the findings of this study may be requested from the correspondent author.

## References

[1] Martin B. C., Warram J. H., Krolewski A. S. (1992). Role of glucose and insulin resistance in development of type 2 diabetes mellitus: results of a 25-year follow-up study. *The Lancet*.

[2] Randrianarisoa E., Lehn-Stefan A., Hieronimus A. (2020). Reduced insulin clearance is linked to subclinical atherosclerosis in individuals at risk for type 2 diabetes mellitus. *Scientific Reports*.

[3] Borai A., Livingstone C., Ferns G. A. (2007). The biochemical assessment of insulin resistance. *Annals of Clinical Biochemistry*.

[4] Sánchez-García A., Rodríguez-Gutiérrez R., Mancillas-Adame L. (2020). Diagnostic Accuracy of the Triglyceride and Glucose Index for Insulin Resistance: A Systematic Review. *International Journal of Endocrinology*.

[5] da Silva A., Caldas A., Rocha D. (2020). Triglyceride-glucose index predicts independently type 2 diabetes mellitus risk: a systematic review and meta-analysis of cohort studies. *Primary Care Diabetes*.

[6] Zhao S., Yu S., Chi C. (2019). Association between macro- and microvascular damage and the triglyceride glucose index in community-dwelling elderly individuals: the Northern Shanghai Study. *Cardio-vasc Diabetol*.

[7] Levey A., Stevens L., Schmid C. (2009). A new equation to estimate glomerular filtration rate. *Annals of Internal Medicine*.

[8] Liu L., Xia R., Song X. (2021). Association between the triglyceride–glucose index and diabetic nephropathy in patients with type 2 diabetes: a cross-sectional study. *Journal of Diabetes Investigation*.

[9] Okamura T., Hashimoto Y., Hamaguchi M., Obora A., Kojima T., Fukui M. (2019). Triglyceride–glucose index is a predictor of incident chronic kidney disease: a population-based longitudinal study. *Clinical and Experimental Nephrology*.

[10] Qi C., Mao X., Zhang Z. (2017). Classification and differential diagnosis of diabetic nephropathy. *Journal of Diabetes Research*.

[11] Schneider F., Saulnier P., Gand E. (2018). Influence of micro- and macro-vascular disease and tumor necrosis factor receptor 1 on the level of lower-extremity amputation in patients with type 2 diabetes. *Cardiovascular Diabetology*.

[12] Paneni F., Beckman J. A., Creager M., Cosentino F. (2013). Diabetes and vascular disease: pathophysiology, clinical consequences, and medical therapy: part I. *European Heart Journal*.

[13] Xu Y., Wu Y., Li J. (2008). The predictive value of brachial-ankle pulse wave velocity in coronary atherosclerosis and peripheral artery diseases in urban Chinese patients. *Hypertension Research*.

